# Assessing attitudes toward research and plagiarism among medical students: a multi-site study

**DOI:** 10.1186/s13010-024-00161-z

**Published:** 2024-11-15

**Authors:** Andrija Pavlovic, Nina Rajovic, Srdjan Masic, Vedrana Pavlovic, Dejana Stanisavljevic, Tatjana Pekmezovic, Dusanka Lukic, Aleksandra Ignjatovic, Miodrag Stojanovic, Dragan Spaic, Nikola Milic, Aleksa Despotovic, Tamara Stanisavljevic, Valerija Janicijevic, Danijela Tiosavljevic, Natasa Milic

**Affiliations:** 1 Department of Humanities, University of Belgrade, Faculty of Medicine, Belgrade, Serbia; 2 Institute for Medical Statistics and Informatics, University of Belgrade, Faculty of Medicine, Belgrade, Serbia; 3 Department of Primary Health Care and Public Health, University of East Sarajevo, Faculty of Medicine, Foca, Bosnia and Herzegovina; 4 Institute of Epidemiology, University of Belgrade, Faculty of Medicine, Belgrade, Serbia; 5 University of Belgrade, Faculty of Medicine, Belgrade, Serbia; 6 Department of Medical Statistics and Informatics, University of Nis, Faculty of Medicine, Nis, Serbia; 7 Department of Biochemistry, University of Belgrade, Faculty of Medicine, Belgrade, Serbia; 8 University of Belgrade, Teacher Education Faculty, Belgrade, Serbia; 9 Mayo Clinic, Rochester, USA

**Keywords:** Research, Plagiarism, Ethics, Questionnaire, Attitudes, Medical education

## Abstract

**Background:**

Research involves the systematic collection and analysis of data to enhance understanding of a particular phenomenon. Participation in medical research is crucial for advancing healthcare practices. However, there has been limited focus on understanding the factors that motivate medical students to engage in research. Additionally, in the era of e-learning, the easy accessibility of online resources has contributed to a widespread ‘copy-paste culture’ among digital-native students, which is recognized in academia as plagiarism. Existing studies suggest that a contributing factor to the increasing prevalence of plagiarism is students’ limited understanding of this act. The purpose of this study was to assess medical students’ attitudes toward research and plagiarism, and to evaluate the psychometric properties of the Attitudes Toward Research (ATR) and Attitudes Toward Plagiarism (ATP) questionnaires.

**Methods:**

This was a multicenter study conducted among medical undergraduate and postgraduate students attending the three medical universities who were involved in research. Students’ attitudes toward research and plagiarism were assessed using the ATR and ATP questionnaires. The research instruments underwent translation and cultural adaptation in accordance with internationally accepted methodology. The psychometric properties of the ATR and ATP, including validity and reliability, were assessed. Confirmatory factor analysis was used to test the model’s fit to the data.

**Results:**

The ATR and ATP questionnaires were completed by 793 medical students who were involved in research (647 undergraduates and 146 PhD students). Cronbach’s alpha coefficients of 0.917 and 0.822 indicated excellent and good scale reliability for the ATR and ATP questionnaires, respectively. The five-and three- factor structures of ATR and ATP have been validated with maximum likelihood confirmatory analysis, and the results demonstrated an adequate level of model fit (TLI = 0.930, CFI = 0.942 and TLI = 0.924, CFI = 0.943, respectively). Medical students showed a high degree of positive attitudes toward research and favorable scores across all three domains of attitudes toward plagiarism. In multivariate regression models, age was found to be positively associated with favorable attitudes of research usefulness, positive attitudes, relevance to life subscales and total ATR scale (*p* < 0.001), while PhD study level was related to research anxiety (*p* < 0.001) and favorable attitudes across all three ATP domains (*p* < 0.001).

**Conclusion:**

Medical students who were involved in research showed a high degree of favorable attitudes toward research and plagiarism. Adjusting medical school curricula to include research courses would broaden the students’ interest in scientific research and maximize their impact on the full preservation of research ethics and integrity.

**Supplementary Information:**

The online version contains supplementary material available at 10.1186/s13010-024-00161-z.

## Introduction

 Research involves the systematic collection and analysis of data to enhance understanding of a particular phenomenon [1]. Its primary goal is to deepen comprehension of the phenomenon under study and disseminate the acquired knowledge to others. Participation in medical research is essential for advancing healthcare practices [2]. Despite a recent comment published in Nature Medicine emphasizing the crucial role of clinician-scientists in the progress of medical research [3], limited attention has been given to understanding how medical students and clinicians can effectively engage in research. Medical students, in particular, face a heavy workload and often lack sufficient time and financial resources [4], which can significantly undermine their motivation to participate in research and pursue academic goals. Assessing students’ attitudes towards research might be a key to understanding students’ needs and identifying gaps in research education [5]. The attitude towards research is defined as an in-depth study of an individual’s thoughts, emotions, and actions in relation to research [6].

Besides problems with medical students involvement in research, an ethical concern has been recognized as a violation of research integrity in contemporary science [7, 8]. Plagiarism, the act of replicating written work or ideas without proper acknowledgment, is commonly referred to as an act of academic misconduct [9]. The problem of plagiarism is not a new occurrence; however, it persists as a substantial challenge that has gained increased public awareness in the last two decades, primarily due to the rapid expansion and widespread availability of digital information. In the era of e-learning, accessing online resources has become the go-to source of knowledge [10]. As a result, plagiarism has evolved into an epidemic, fostering a widespread “copy-paste culture” among students from the digital native generation [11]. Existing research suggests that the increasing prevalence of plagiarism is due to students’ limited comprehension of this act, a tendency among students to engage in such behavior, and a lack of awareness of the severity of these violations. Understanding students’ attitudes toward plagiarism may be critical in preventing plagiarism [12–14].

Researchers have developed several reliable tools [13, 15–20] for assessing attitudes toward research and plagiarism. The Attitudes Toward Research (ATR) [15, 21] and Attitudes Toward Plagiarism (ATP) [13] questionnaires are relevant and standardized instruments for assessing the attitudes of different populations towards research and plagiarism. Clear, systematic scales design the instruments, facilitating straightforward data interpretation and consistency across responses. Therefore, the aim of this study was to assess attitudes towards research and plagiarism among undergraduate and postgraduate medical students, to evaluate the psychometric properties of the ATR and ATP questionnaires, and to correlate the students’ attitudes toward research and plagiarism with their personal characteristics and educational background.

## Materials & methods

### Study design

This was a multicentre study conducted during the 2022–2023 school year among medical undergraduate and postgraduate students attending the three medical universities in the Western Balkans: the University of Belgrade Faculty of Medicine, the University of Niš Faculty of Medicine, and the University of East Sarajevo Faculty of Medicine. The inclusion criterion for participation in the study was a completed undergraduate or postgraduate course in scientific research (details provided in Table 1). The ATR and ATP questionnaires were distributed to all medical students fulfilling inclusion criteria. The response rate was 93.3% (793/850).

**Table 1:** Study population: list of completed research courses

	University of Belgrade		University of Niš	University of East Sarajevo
**Study level**	Undergraduate studies	PhD studies	Undergraduate studies	Undergraduate studies
**Completed course**	Scientific research	Informatics for researchers	Scientific research	Methodology in Scientific Research

Course completion was a prerequisite for active participation in medical student conferences. The curriculum for undergraduate students included an introduction to science and research, ethical concepts, types of research, the structure of original scientific work, how to write a paper, and how to cite literature. The postgraduate course combined the core principles of research methodology with basic biomedical informatics terminology and statistical methods necessary for critical data analysis in a cross-disciplinary setting. We delivered all courses in a blended (hybrid) mode, giving students access to a continuous learning environment that included essential materials, video lectures, informative presentations, formative assessments through quizzes, and summative evaluations through tests. Demographic and educational background data were collected for all participants. For students who wrote scientific papers and attended conferences, additional categorization included scientific field of interest (preventive/preclinical vs. clinical). Data were collected online using a distance learning platform.

The research was conducted in accordance with the principles of good research practice, ensuring voluntary participation and full preservation of data confidentiality. Written informed consent was obtained from all participants in the study. The authors did not have access to information that could identify individual participants during or after data collection. The University of Belgrade Faculty of Medicine’s Ethics Committee approved the study (Ethical Code: 25/VII-10).

### Study instruments

#### 1. Attitudes towards research (ATR) scale

Attitudes towards research were determined using the ATR scale [22]. Elena C. Papanastasiou, the author of the questionnaire, provided consent before conducting the study. The ATR scale contains 30 items scored on a seven-point Likert scale, where 1 indicates strong disagreement and 7 indicates strong agreement. There are five subscales within the ATR scale: research usefulness for the profession (9 items), research anxiety (6 items), positive attitudes towards research (7 items), relevance to life (4 items), and research difficulty (2 items). Prior to data analysis, negatively worded items were reverse-scored so that a higher Likert scale score could indicate positive attitudes. The mean score for each subscale was determined by adding the scores for the subscale items and dividing by the number of items within the subscale.

#### 2. Attitudes toward plagiarism (ATP) questionnaire

The ATP questionnaire was used to assess students’ attitudes toward plagiarism [13]. Prior to the study’s commencement, we obtained formal authorization from the questionnaire developer, who granted permission for the instrument’s validation and utilization. The ATP questionnaire consists of 29 statements grouped into three domains: positive attitude, negative attitude, and subjective norms. Statements are graded using a five-point Likert scale: 1 (strongly disagree), 2 (disagree), 3 (neither agree nor disagree), 4 (agree), and 5 (strongly agree). According to the Likert scale, points are allocated to each answer, and the scores of domains are computed based on their sum. The positive attitude domain indicates approval and justification for plagiarism. This domain consists of 12 statements with a scoring range of 12 to 60, with lower values indicating favorable attitudes. The statements assessing individuals’ positive attitude towards plagiarism primarily relate to the actions undertaken by the participants themselves. A negative attitude towards plagiarism signifies disapproval and condemnation of this behavior. The domain consists of seven statements, each of which is assigned a score ranging from 7 to 35, with higher values indicating favorable attitudes. Negative attitudes are typically associated with procedures performed by others or societal norms in general. Subjective norms refer to an individual’s personal perception of the prevalence and societal acceptance of plagiarism. This domain consists of 10 statements, each assigned a score ranging from 10 to 50, where lower values present favorable attitudes. Participants who possess low subjective norms towards plagiarism tend to view such behavior as socially unacceptable [13]. According to Pupovac et al. [23], all domain scores are divided into low, moderate, and high score categories. Favorable attitudes from the academic integrity point of view are as follows: The positive attitude score ranging from 12 to 28 suggests that individuals possess a low level of tolerance towards acts of plagiarism; the negative attitude score ranging from 27 to 35 signifies a strong disapproval of plagiarism, reflecting an attitude that does not tolerate any form of academic dishonesty; and the subjective norm score ranging from 10 to 23 indicates that one tends to view such behavior as socially unacceptable.

### Translation and cultural adaptation

The research instruments underwent translation and cultural adaptation in accordance with internationally accepted methodology [24]. The initial iteration of the questionnaire validation was translated into Serbian by two independent translators, both of whom have Serbian as their native language. To ensure a translation that closely resembles the original instrument, one translator had prior knowledge of the concepts intended to be assessed by the questionnaire, while another translator approached the task with a lack of familiarity, aiming to generate a translation in which any minor differences may be found. The initial translators thoroughly examined and resolved the discrepancies. In order to ensure the translation’s accuracy, the next phase involved the back-translation of the instrument by two independent English translators who were native speakers fluent in Serbian. In order to avoid bias, the back-translators were deliberately kept unaware of the specific topics that the questionnaire aimed to assess. A comparative analysis was conducted between the original text and the back-translated version, and any discrepancies were resolved through consensus among members of an expert committee. Individuals with expertise in methodology, knowledge of the construct of interest, and experience in both forward and backward translation comprised the expert committee.

### Statistical analysis

Descriptive statistics are presented as frequencies and percentages for categorical variables and means and standard deviations for continuous variables. The sample size was determined using the set criteria for factor analysis, which stipulates a minimum of 150 respondents and a minimum of 5 participants for each item. However, the study included all students enrolled in scientific research at three universities (*n* = 793). The analysis did not use any imputation methods for missing data. We automated the scoring system of these instruments, which enhanced efficiency by enabling quick and accurate data processing. This contributed to this study’s effective and pragmatic approach to instrument selection. The psychometric properties of the ATR and ATP were evaluated, including validity and reliability. Confirmatory factor analysis (CFA) was conducted using the maximum likelihood estimation method to assess the model’s fit to the data. The absolute goodness-of-fit of the models was evaluated using the chi-square test, with values less than 0.05 indicating a poor fit for the data. We also used the comparative fit index (CFI), the incremental fit index (IFI), and the root mean square error of approximation (RMSEA) to assess the model fit. Values of CFI and IFI above 0.90 were considered adequate, whereas RMSEA values below 0.06 indicated an acceptable model fit. The internal consistency for the entire scale and its subscales was assessed using Cronbach’s coefficient. Scales with an alpha coefficient equal to or greater than 0.70 were considered acceptable [25]. The Student’s t test and one-way ANOVA were used to assess differences between groups. Univariate and multivariate linear regression analyses were used to identify factors associated with students’ attitudes towards research. Univariate and multivariate logistic regression analyses were performed to assess predictors of favorable attitudes for ATP domains. All tests were two-tailed. *P* < 0.05 was considered statistically significant. Statistical analysis was done using Amos 21 (IBM SPSS Inc., Chicago, IL, 2012) and IBM SPSS Statistics 25 software.

## Results

793 medical students (647 undergraduate medical students and 146 PhD students) completed the ATR and ATP questionnaires. The average age of study participants was 24.1 ± 4.5 years (range: 18–55) and predominantly female (68.5%). Table 2 presents the demographic characteristics and educational background of the study participants.

**Table 2:** Demographic characteristics and educational background of study participants

Variable	Total (*n* = 793)	University of Belgrade (*n* = 369)	University of Niš (*n* = 177)	University of East Sarajevo (*n* = 101)	University of Belgrade (PhD) (*n* = 146)
Gender, n (%)					
Male	247 (31.5)	121 (32.8)	49 (29.0)	30 (29.7)	47 (32.4)
Female	537 (68.5)	248 (67.2)	120 (71.0)	71 (70.3)	98 (67.6)
Age, mean ± sd	24.1 ± 4.5	23.3 ± 1.2	23.0 ± 1.4	20.0 ± 0.9	31.0 ± 6.9
GPA, mean ± sd	9.2 ± 0.6	9.2 ± 0.6	9.2 ± 0.6	NA	NA
Study year					
I	101 (12.9)	0 (0.0)	0 (0.0)	101 (100.0)	NA
II-VI	546 (68.9)	369 (100.0)	177 (100.0)	0 (0.0)	
Scientific field,					
Preventive/Preclinical	203 (40.4)	143 (38.8)	60 (45.1)	NA	NA
Clinical, n (%)	299 (59.6)	226 (61.2)	73 (54.9)		

The average ATR total score was 5.1 ± 0.8 (range 1–7), indicating positive attitudes of medical students towards research. Favorable average ATP scores were obtained for all three domains of plagiarism (positive attitudes, negative attitudes, and subjective norms: 2.4 ± 0.7, 3.9 ± 0.7, and 2.3 ± 0.7, respectively) (Table 3). Students who perceived research as useful, held positive attitudes toward research, regarded research as relevant to life, and found research challenging exhibited favorable attitudes in the positive attitudes, negative attitudes and subjective norms domains of the ATP questionnaire. Students who experienced research anxiety showed favorable attitudes in Positive Attitudes, and Subjective norms domains (Table 4).

**Table 3:** ATR and ATP scores among undergraduate and postgraduate medical students

ATR		ATP		
Domain	mean ± sd	Domain	mean ± sd	
Research usefulness	5.8 ± 1.1	Positive attitudes		2.4 ± 0.7
Research anxiety	3.7 ± 1.2	Negative attitudes		3.9 ± 0.7
Positive attitudes	5.5 ± 1.2	Subjective norms		2.3 ± 0.7
Relevance to life	5.0 ± 1.0	Total ATP		NA
Difficulty of research	4.9 ± 1.3			
Total ATR	5.1 ± 0.8			

**Table 4:** Correlations between ATR and ATP domains

	1.	2.	3.	4.	5.	6.	7.	8.
**ATR domains**								
1. Research usefulness								
2. Research anxiety	-0.106*							
3. Positive attitudes	0.843**	0.013						
4. Relevance to life	0.593**	0.067	0.624**					
5. Difficulty of research	0.194**	0.518**	0.285**	0.321**				
6. Total ATR	0.827**	0.381**	0.880**	0.719**	0.538**			
**ATP domains**								
7. Positive attitudes	-0.296**	-0.105*	-0.296**	-0.340**	-0.334**	-0.369**		
8. Negative attitudes	0.422**	-0.056	0.388**	0.340**	0.189**	0.385**	-0.511**	
9. Subjective norms	-0.337**	-0.138**	-0.356**	-0.361**	-0.414**	-0.433**	0.713**	-0.524**

According to ATP categorization, almost half (45.4%) of the students had favorable positive attitudes, 58.9% had favorable negative attitudes, and 65.3% had favorable subjective norms (Additional file 1).

The frequency of students’ responses to ATR scale items is presented in Additional file 2. Most (85.6%) of students agreed that research is useful for their career, 84% considered research skills to be helpful in the future, 80.4% suggested incorporating research into the undergraduate curriculum, and 73.6% suggested incorporating it into professional training. Almost half answered that research makes them anxious (45.8%) and nervous (37.7%), although only 16.1% considered research a complex and difficult (19.8%) subject. Regarding the positive attitudes subscale, 77.5% enjoyed research, and 74.2% agreed that students benefit from research. More than half (64.4%) agreed that research-oriented thinking plays an important role in everyday life, and 39.6% stated they use research in their daily lives. The majority (68.5%) agreed that research concepts are difficult to understand, and almost half (47.8%) were of the opinion they made mistakes during the research (Additional file 2).

Additional file 3 presents the distribution of students’ answers to all ATP statements. In the positive attitudes domain, more than a third, 38.1% of participants, agreed that using others’ words without citation can sometimes be unavoidable, while 8.2% strongly disagreed. Opinions on self-plagiarism varied, with 19.2% agreeing that it is not punishable and 15.1% strongly agreeing, whereas 13.1% firmly disagreed. When considering the punishment for self-plagiarism, 33.3% agreed it should not be punishable in the same way as plagiarism, with 9.3% strongly disagreeing. When considering the negative attitudes towards plagiarism, 37.3% agreed that plagiarists do not belong in the scientific community, and 27.7% strongly agreed. A significant proportion (82.4%) of students expressed the belief that engaging in discussions on topics such as plagiarism and self-plagiarism is of great importance. The majority of students (78.3%) also agreed that plagiarism has a detrimental effect on the spirit of research and investigation. Additionally, 31.5% agreed that a plagiarized paper does not harm science, but 42.5% strongly disagreed. Subjective norms toward plagiarism indicate skepticism about the honesty of others, with 56.1% neither agreeing nor disagreeing that authors who claim not to plagiarize are truthful. Furthermore, 58.8% strongly disagree with the idea that they persist in plagiarizing due to the lack of detection. The perception of the environment varies, as 45.0% neither agree nor disagree that they work in a plagiarism-free setting (Additional file 3).

The internal consistency analysis of the Serbian version of the ATR questionnaire yielded a Cronbach’s alpha of 0.917 for the entire scale, which indicates excellent scale reliability (Table 5). The subscales ranged in reliability from 0.94 (positive attitudes subscale) to 0.61 (relevance to life subscale). Cronbach’s alpha coefficient of the overall 29-item ATP questionnaire was 0.822, which indicates that the scale has good reliability. The Cronbach’s alpha coefficients of the positive attitude domain, negative attitude domain, and subjective norm domain were 0.887, 0.791, and 0.884, respectively (Table 5).

**Table 5:** Average scores and reliability statistics

Measure	Cronbach´s Alpha	Internal Consistency
ATR		
Research usefulness	0.933	Excellent
Research anxiety	0.850	Good
Positive attitudes	0.939	Excellent
Relevance to life	0.606	Questionable
Difficulty of research	0.653	Questionable
Total ATR	0.917	Excellent
**ATP**		
Positive attitudes	0.887	Good
Negative attitudes	0.791	Acceptable
Subjective norms	0.884	Good
Total ATP	0.822	Good

**Fig. 1:**
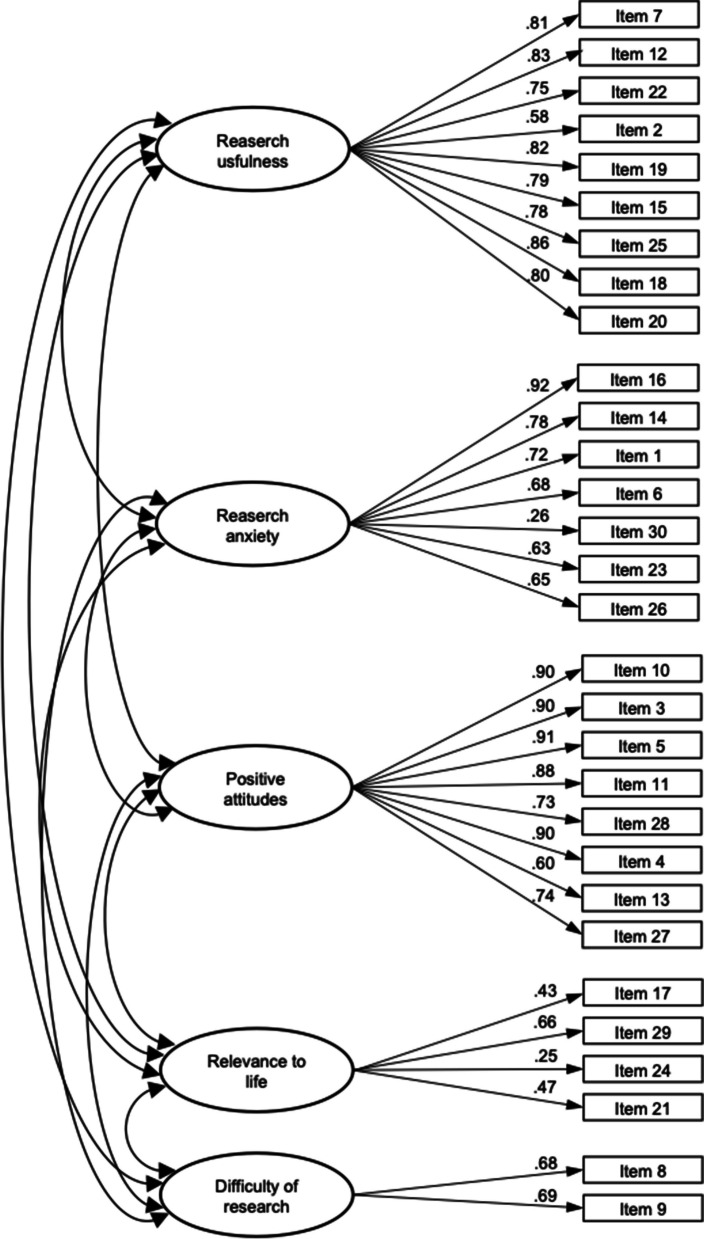
Standardized factor loadings for the Serbian version of ATR scale

The five-factor structure of ATR was assessed by conducting a confirmatory factor analysis. We used the maximum likelihood method in CFA. The chi-square test rejected the five-dimensional model, as expected, since this measure of fit is known to be sensitive to sample size (χ2 = 1368,041, df = 361, *p* < 0.001). The values for fit indices TLI (0.930) and CFI (0.942) were above their cut-off criteria, indicating an acceptable level of model fit. The RMSEA value of 0.059 (0.056–0.063) was below the suggested value of 0.06, also suggesting adequate fit. Standardized factor loadings were statistically significant and ranged from 0.25 to 0.92 (Fig. 1).

**Fig. 2:**
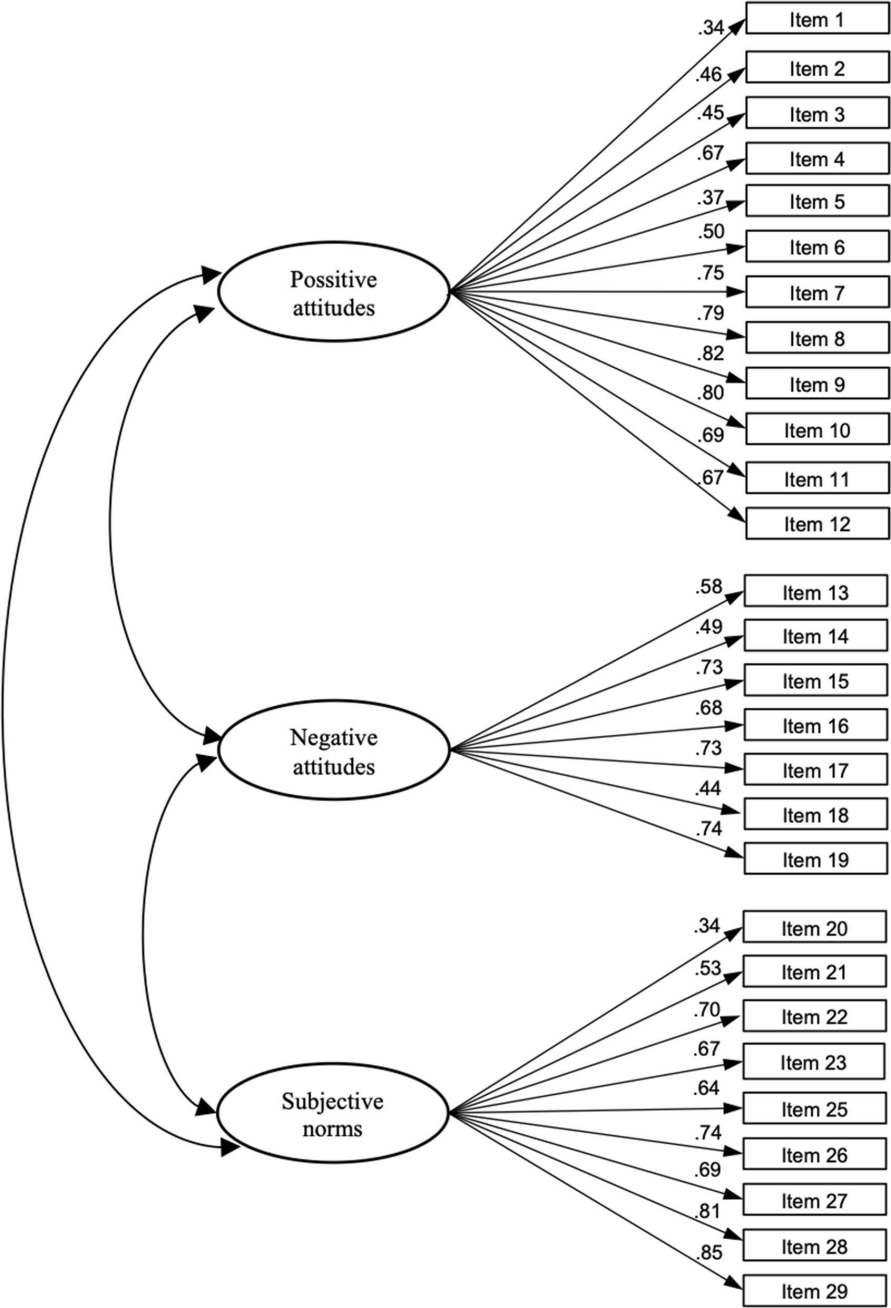
Standardized factor loadings for the ATP questionnaire in the Serbian language

Maximum likelihood confirmatory analysis validated the three-factor structure of the ATP questionnaire, and the results showed an adequate level of model fit. The chi-square test rejected the three-dimensional model, as was expected due to the large sample size (χ2 = 922.460, df = 282, *p* < 0.001). The values for fit indices TLI (0.924) and CFI (0.943) were close to their cut-off criteria. The RMSEA value of 0.054 (CI 0.050–0.057) was below 0.06, indicating an acceptable model fit. Standardized factor loadings were statistically significant and ranged from 0.34 to 0.85. Figure 2 presents the standardized factor loadings for the ATP questionnaire in the Serbian language.

 Female students had a higher level of research anxiety than male students. Nearly all subscales and total ATR showed a correlation with age and GPA. In general, PhD students and II-VI-year students had more favorable attitudes toward research (total ATR), considered research more useful, and had higher scores on the positive attitudes subscale than freshmen students. PhD students scored higher on the relevance of life subscale compared to II-VI year and freshmen students, and II-VI year students were more favorable than freshman students. PhD students had a higher level of research anxiety than II-VI year and freshmen students, and II-VI-year students compared to freshman students. II-VI year students considered research less difficult than PhD and freshman students, and PhD students compared to freshman students. Students involved in preclinical research had higher scores on the positive attitudes subscale than students involved in clinical research (Table 6).

**Table 6:** Attitudes toward research and plagiarism among medical students

*n* = 793	ATR						ATP		
	Research usefulness	Research anxiety	Positive attitudes	Relevance to life	Difficulty of research	Total ATR	Positive attitudes	Negative attitudes	Subjective norms
Gender									
Male	5.8 ± 1.0	3.8 ± 1.1	5.6 ± 1.1	5.0 ± 1.1	4.9 ± 1.3	5.1 ± 0.8	2.45 ± 0.73	3.97 ± 0.66	2.30 ± 0.74
Female	5.8 ± 1.1	3.6 ± 1.2*	5.5 ± 1.2	4.9 ± 1.0	4.8 ± 1.3	5.0 ± 0.8	2.44 ± 0.75	3.88 ± 0.69	2.33 ± 0.72
Age, r	0.139*	-0.102*	0.157*	0.184*	-0.005	0.114*	-0.244*	0.185*	-0.127*
GPA, r	0.158*	0.086*	0.193*	0.163*	0.138*	0.209*	-0.132*	0.132*	-0.091*
Study level									
I	4.8 ± 1.4^a, b^	4.1 ± 1.3 ^a, b^	4.2 ± 1.5 ^a, b^	4.2 ± 1.0 ^a, b^	4.2 ± 1.6 ^a, b^	4.3 ± 0.9 ^a, b^	3.17 ± 0.74^a, b^	3.42 ± 0.80^a, b^	3.01 ± 0.81^a, b^
II-VI	5.9 ± 1.0	3.7 ± 1.2 ^c^	5.7 ± 1.0	5.0 ± 0.9 ^c^	5.0 ± 1.2 ^c^	5.2 ± 0.7	2.38 ± 0.68^c^	3.93 ± 0.65^c^	2.20 ± 0.66
PhD	6.0 ± 0.8	3.4 ± 1.1	5.8 ± 0.8	5.3 ± 1.0	4.7 ± 1.3	5.2 ± 0.6	2.17 ± 0.63	4.14 ± 0.55	2.25 ± 0.64
Preventive/Preclinical	5.9 ± 1.0	3.7 ± 1.2	5.8 ± 1.0*	5.1 ± 0.9	5.2 ± 1.1	5.2 ± 0.7	2.34 ± 0.70	3.98 ± 0.67	2.10 ± 0.64*
Clinical	5.9 ± 0.9	3.7 ± 1.2	5.6 ± 0.9	5.0 ± 1.0	5.0 ± 1.2	5.1 ± 0.7	2.39 ± 0.67	3.89 ± 0.61	2.25 ± 0.64
*p<0.050 aI vs II-VI *p*<0.001; bI vs PhD *p*<0.001; cII-VI vs PhD *p*<0.00									

Data are presented as mean±sd, r-Pearson correlation coefficient

There were no differences in ATP domain scores according to gender. All three domain scores showed a correlation with age and GPA. PhD students exhibited more favorable average scores for both positive and negative attitudes compared to II-VI year and freshmen students, while II-VI year students were more favorable compared to freshman students. Compared to freshman students, PhD and II-VI year students had more favorable average scores for the subjective norm domain. On average, students in the preclinical field had more favorable attitudes toward the subjective norm domain than students in the clinical research field (Table 6). The scores of attitudes toward research and plagiarism according to study level are presented in Additional file 4, Additional file 5 and Additional file 6. Among II-VI year students, results showed a significant gender difference in research anxiety, with female students reporting higher levels compared to male students. Correlation analyses revealed significant positive relationships between GPA and all of the domains of both ATR and ATP questionnaire. Students in the Preventive/Preclinical field exhibited more favorable positive attitudes towards research and subjective norms compared to their Clinical counterparts (Additional file 5). Among PhDs, male students reported lower research usefulness and showed higher positive attitudes toward plagiarism than female students (Additional file 6).

In univariate regression analysis, PhD study level and age were positively associated with favorable attitudes toward research usefulness, research anxiety, positive attitudes, and relevance to life subscales. Gender was associated with research anxiety, whereas PhD was negatively associated with research difficulty. Age was associated with overall attitudes toward research (total ATR). In multivariate linear regression models, older age was linked to more positive attitudes about research usefulness, positive attitudes, relevance to life subscales, and the total ATR scale. On the other hand, the level of study for a PhD was linked to research anxiety, showing that PhD students have a higher level of anxiety (Table 7). A subgroup analysis of undergraduate students who attended the students’ conference revealed a positive association between GPA and attitudes on all five subscales and the total ATR scale, and a positive association between the preclinical scientific field and favorable attitudes on the positive attitudes subscale (Additional file 7).

**Table 7:** Univariate and multivariate linear regression analysis among undergraduate and post graduate medical students with total ATR and ATR subscales as dependent variables

ATR subscales *n* = 793	Univariate			Multivariate		
	β	t	*p*	β	t	*p*
**Research usefulness**						
Gender, male	-0.001	0.041	0.967			
Age	0.139	3.890	**< 0.001**	0.139	3.890	**< 0.001**
Study level, PhD	0.111	3.154	**0.002**			
**Research anxiety**						
Gender, male	0.072	2.014	**0.044**			
Age	-0.102	2.841	**0.005**			
Study level, PhD	-0.120	3.400	**< 0.001**	-0.128	3.559	**< 0.001**
**Positive attitudes**						
Gender, male	0.046	1.287	0.199			
Age	0.157	4.406	**< 0.001**	0.157	4.406	**< 0.001**
Study level, PhD	0.103	2.918	**0.004**			
**Relevance to life**						
Gender, male	0.021	0.576	0.564			
Age	0.184	5.193	**< 0.001**	0.184	5.193	**< 0.001**
Study level, PhD	0.149	4.249	**< 0.001**			
**Difficulty of research**						
Gender, male	0.046	1.278	0.202			
Age	-0.005	0.138	0.891			
Study level, PhD	-0.073	2.053	**0.040**			
**Total ATR**						
Gender, male	0.051	1.436	0.152			
Age	0.114	3.180	**0.002**	0.114	3.180	**0.002**
Study level, PhD	0.062	1.741	0.082			

Table 8 presents univariate and multivariate logistic regression analyses with attitudes toward plagiarism as dependent variables. In univariate logistic regression analysis, age and study level were predictors of favorable attitudes across all three ATP domains (*p*<0.001). In multivariate logistic regression models, study level was a significant independent predictor of attitudes toward plagiarism for all three ATP domains (*p*<0.001). PhD status and II–VI year of study were associated with favorable attitudes across all three ATP domains (*p*<0.001). In a subgroup analysis of undergraduate students who participated at the students' conference, GPA was a predictor of favorable attitudes toward plagiarism across all three ATP domains in univariate logistic regression analysis (*p*<0.05). Higher GPA (*p*=0.048) and interest in preclinical or preventative scientific fields (*p*=0.019) were found to be independent predictors of positive attitudes in the subjective norms domain (Additional file 8).

**Table 8:** Univariate and multivariate logistic regression analysis among undergraduate and post graduate medical students with ATP subscales as dependent variables

Variable	Univariate			Multivariate		
	*p*	OR	95%CI	*p*	OR	95%CI
**Positive attitudes**						
Gender	0.426	0.884	0.653 − 0.197			
Age	**< 0.001**	1.087	1.046–1.130			
Study level						
I	Ref.					
II-VI	**< 0.001**	4.235	2.449–7.324	**< 0.001**	4.223	2.441–7.307
PhD	**< 0.001**	8.175	4.400-15.190	**< 0.001**	7.751	4.134–14.532
**Negative attitudes**						
Gender	0.067	1.337	0.980–1.823			
Age	**< 0.001**	1.121	1.069–1.177			
Study level						
I	Ref.					
II-VI	**< 0.001**	3.793	2.385–6.032	**< 0.001**	3.765	2.366–5.991
PhD	**< 0.001**	7.056	3.999–12.451	**< 0.001**	7.373	4.110-13.225
**Subjective norms**						
Gender	0.746	1.054	0.767–1.447			
Age	**< 0.001**	1.106	1.053–1.162			
Study level						
I	Ref.					
II-VI	**< 0.001**	8.241	5.024–13.517	**< 0.001**	6.752	3.967–11.493
PhD	**< 0.001**	7.201	4.042–12.827	**< 0.001**	3.800	1.645–8.778

## Discussion

In this multicenter study, most medical students who were involved in research showed a high degree of favorable attitudes toward research and plagiarism. A Serbian version of the ATR and ATP questionnaires was validated among undergraduate and PhD medical students, and the study revealed a satisfactory level of both instruments’ validity and reliability within the Serbian educational context.

Medical research serves as the driving force behind advancements in healthcare. Its dynamic nature, extensive scope, and continuous growth encourage the development of novel clinical practices. However, effectively translating these discoveries into clinical practice relies heavily on the active participation of future healthcare professionals. Undergraduate students often find research method courses complicated and overwhelming, leading to feelings of anxiety, and misconceptions about research [26]. Understanding student attitudes towards research is therefore critical for teachers to foster positive attitudes towards research and implement effective strategies for learning research methods. Several validation studies evaluated the psychometric properties of the ATR questionnaire, often used to assess students’ attitudes toward research. Researchers found it to be a useful tool for understanding students’ perspectives on research [5, 27–30]. According to our results, Serbian medical students held favorable attitudes toward research. The results of previous studies are inconsistent, ranging from negative and neutral [11, 15, 19, 22] to positive students’ attitudes toward research [10, 20, 23].

The results of this study also show differences in attitudes towards research with regard to students’ study levels. PhD students and II-VI students had the highest total ATR scores, while first-year students had the lowest score. Scores for research usefulness, positive attitudes, and relevance to life were highest among PhD students, except for research anxiety. All first-year PhD students are required to take the “Informatics for Researchers in Medicine” course, and the need to publish research articles to complete their studies influences their attitudes, leading to the expected results. PhD students held favorable attitudes toward research, unlike first-year students who attended an introductory course in research methodology but still haven’t decided on their career path. These results are consistent with previous studies, showing more positive attitudes towards research by students of higher years of studies and PhD students [31, 32], but considering research more stressful than freshman students [33]. According to our results, students involved in preclinical research have more positive attitudes toward research than students involved in clinical research. We should make potential changes in research courses to better facilitate clinical study groups, given the discrepancy between these student groups.

The pressure to continuously publish for career advancement is placing academics under significant strain, leading to the emergence of another issue in research [34]. Inexperienced researchers and students, facing the “Publish or Perish” imperative, are at risk of turning to unethical practices such as plagiarism due to a lack of familiarity with ethical guidelines pertaining to publication [35]. Whether it is accidental or on purpose, the consequences of unethical behavior are certain, affecting not only medical researchers but also students who engage in such practices. The scientific community is responsible for determining the underlying factors contributing to plagiarism, with the aim of developing effective preventative measures. Investigating attitudes toward plagiarism at the early stages of education is crucial for the success of those measures [7].

The ATP questionnaire is based on Ajzen’s theory, positing that planned behavior can effectively predict an individual’s anticipated actions through attitudes and intentions [13]. In our study, students had on average favorable attitude scores across all three domains of attitudes toward plagiarism. A recent study on 551 students who were provided with an ATP questionnaire revealed average scores for positive attitudes, negative attitudes, and subjective norms toward plagiarism of 31.34 ± 7.26, 25.26 ± 4.61, and 25.16 ± 6.12, respectively [36]. The scores indicated that students enrolled in Saudi Arabian medical institutes have moderate attitudes towards plagiarism. Another study, conducted in Croatia, assessed attitudes towards plagiarism among students studying medical biochemistry and pharmacy [23]. It also found moderate scores for all three scales, indicating a significant proportion of students tolerated and rationalized the act of plagiarism. Furthermore, the study found that 59% of students thought plagiarism was harmless, 63% thought it was not very important, and 35% considered it was sometimes necessary [23].

The latest study by Phyo et al. investigated attitudes, self-reported practices, and knowledge regarding plagiarism in postgraduate medical students [37]. A significant proportion of students considered plagiarism acceptable and justifiable. Furthermore, more than one-third of participants engaged in at least one act of plagiarism. A third of the respondents expressed disagreement with the statement that self-plagiarism is not subject to punishment due to its lack of harm (since one cannot take from oneself). More than half (58.2%) of students expressed disagreement with the statement “I could not write a scientific paper without plagiarizing,” and only 35.7% disagreed with plagiarizing practices [37]. Similarly to these results, our study revealed that 32.1% disagreed with the statement that self-plagiarism is not punishable due to its lack of harm. Furthermore, 77.1% of students agreed that plagiarism is not a serious offence and only 36.7% stated that they studied/worked in a plagiarism-free environment. However, 77.8% of our study population disagreed that they could not write a scientific paper without plagiarizing, and 63.3% disagreed with the statement that sometimes it is necessary to plagiarize. Our study population’s prior experience in scientific research and their enrollment in introductory research courses from the first year of their medical education could explain this. A study on 150 medical students from Romania’s University of Medicine and Pharmacy of Craiova revealed a predominant acceptance of plagiarism [38]. Students attending both private and public medical schools in Pakistan showed similar results [39]. More than half (55%) of medical students endorsed academic dishonesty.

Our study shows significant differences in attitudes toward plagiarism depending on the level of study. Freshman students showed, on average, less favorable positive attitudes than students attending the II–VI school year and PhD students. At the earliest stage of medical education, freshman students have a limited understanding of the concept of plagiarism, its consequences, and the importance of academic integrity. They are unaware of what constitutes plagiarism or how to properly cite sources, as demonstrated in our results. Attitudes become more favorable as students move toward higher levels of education, but expectations and pressure to perform become increasingly present. Under such pressure, students with higher education levels can, in fact, present more unfavorable attitudes toward plagiarism. The Tehran University of Medical Sciences study, involving 230 clerkship, internship, and residency students, illustrates this paradox by revealing a limited understanding of plagiarism among residents compared to younger students [40]. In addition, in our study, gender was associated with differences in attitudes towards plagiarism with females having lower positive attitudes towards plagiarism which is in accordance with previously published study [41].

Although prevalent, research misconduct can be influenced by a range of other factors, such as cultural background, campus culture, or academic discipline. Cultural norms significantly influence students’ perceptions of academic integrity and dishonesty. Individuals in societies that focus on the “group” rather than the individual frequently prioritize the dissemination of information over the possession of personal ideas. Students from such countries may originate from educational systems that prioritize rote memorization and collaboration, in contrast to countries whose academic standards promote individual originality and intellectual property [42]. In addition, insufficient training in scientific writing along with publication pressure, the lack of clear understanding of plagiarism and the cultural tolerance of unethical conduct prevalent in numerous South, East, and Southeast Asian nations may contribute to existing differences in attitudes toward plagiarism [43]. Various academic disciplines also affect students’ perceptions of plagiarism. Disciplines such as engineering, where collaboration and knowledge exchange are essential to learning, may cultivate a more permissive stance on plagiarism in contrast to fields like the humanities, where originality in writing is critical. A study comparing engineering and humanities students revealed that engineering students were more inclined to rationalize cheating, perceiving it as a strategy to manage substantial workloads or achieve collective objectives [44]. Recently published study [45] indicated that all academic disciplines focus on teaching students proper citation practices, however, technical fields, natural and health sciences place less emphasis compared to social sciences. The study results also concluded that education reduces plagiarism, but to fully address the issue, further preventive or punitive measures are needed. Despite their awareness of the consequences, students are more likely to engage in plagiarism due to the frequent absence of repercussions. This suggests that educational initiatives on plagiarism serve primarily as a supplement to punitive measures designed to discourage students from engaging in such behaviors. In Serbia, The Code of Academic Integrity for Higher Education Institutions in Serbia, adopted by the National Council for Higher Education in 2016, and The Code of Scientific Research adopted by National Council for Scientific and Technological Development of the Ministry of Science, Innovation and Technological Development set standards of academic integrity and procedures for determining non-academic behavior. All research institutions, faculties, and universities should satisfy the fundamental standards of integrity, while establishing a unique code that incorporates specific elements relevant to their work.

Educating both undergraduate and PhD medical students according to suggested codes can properly develop students’ attitudes regarding research and plagiarism at the early beginning of their career as medical professionals and sustain them as they advance in their education. Research demonstrates that gaining knowledge about research fosters positive attitudes towards health research, which is essential for enhancing health care [46]. To ensure the rigor of medical research in the academic setting, we should implement a comprehensive institutional policy that promotes research, prohibits plagiarism, and implements a rigorous system of disciplinary measures.

## Strengths and limitations

The strength of our study lies in its multi-site approach involving three medical universities in the Western Balkans, as well as the high response rate. However, several limitations should be acknowledged. Firstly, our sample consisted mostly of female respondents. This phenomenon can be attributed to the higher enrollment rate of female students in the Faculty of Medicine in this region. Secondly, we have included only PhD students from one university. Additionally, there was the presence of self-selection bias, potentially resulting in an underestimation of self-reported plagiarism practices. Lastly, we depended on participants’ self-reported practices, which may underrepresent the actual prevalence of research misbehaviors stemming from participants’ tendencies to provide socially desirable responses.

## Conclusions

The present study revealed ATR and ATP as reliable and valid tools for identifying medical students’ attitudes towards research and plagiarism within the Serbian educational context. The ATR and ATP scales’ five-factor (research usefulness for the profession, research anxiety, positive attitudes towards research, relevance to life, and research difficulty) and three-factor (positive attitudes, negative attitudes, and subjective norms) structures were validated by confirmatory factor analysis, respectively. Overall, medical students showed a high degree of positive attitudes toward research and favorable scores across all three domains of attitudes toward plagiarism. More favorable scores observed in PhD students support the implementation of educational interventions at the early stages of their academic careers aimed at promoting medical research and avoiding plagiarism. Adjusting medical school curricula to include research courses would broaden the students’ interest in scientific research and maximize their impact on the full preservation of research ethics and integrity.

## Supplementary Information

Additional file 1. Categorized ATP domain scores of study participants.

Additional file 2. Participants’ responses to ATR questionnaire.

Additional file 3. Participants’ responses to ATP questionnaire.

Additional file 4. Attitudes toward research and plagiarism among first year students.

Additional file 5. Attitudes toward research and plagiarism among II-VI year students.

Additional file 6. Attitudes toward research and plagiarism among PhD students.

Additional file 7. Univariate and multivariate linear regression analysis of undergraduate students who participated at students’ conference with ATR subscales as dependent variables.

Additional file 8. Univariate and multivariate logistic regression analysis of undergraduate students who participated at students’ conference with ATP domains as dependent variable.

## Data Availability

No datasets were generated or analysed during the current study.
