# Supplementary material for: Assessing attitudes toward research and plagiarism among medical students: a multi-site study
Source: Philos Ethics Humanit Med. 2024 Nov 15;19:11. doi: 10.1186/s13010-024-00161-z (PMC11566133; doi:10.1186/s13010-024-00161-z)
Supplement: Supplementary file 1 — Additional file 1. Categorized ATP domain scores of study participants. [file 13010_2024_161_MOESM1_ESM.docx]

**Table** Categorized ATP domain scores of study participants

|  | **Positive attitudes** | | | **Negative attitudes** | | | **Subjective norms** | | |
| --- | --- | --- | --- | --- | --- | --- | --- | --- | --- |
|  | **Low*** | **Moderate** | **High** | **Low** | **Moderate** | **High*** | **Low*** | **Moderate** | **High** |
| **Total** | 360 (45.4) | 396 (49.9) | 37  (4.7) | 10  (1.3) | 316 (39.8) | 467 (58.9) | 518 (65.3) | 267 (33.7) | 8  (1.0) |
| **Gender** |  |  |  |  |  |  |  |  |  |
| Male | 107 (30.1) | 130 (33.2) | 10 (27.0) | 2  (20.0) | 88  (28.1) | 157 (34.1) | 163 (31.9) | 80  (30.2) | 4  (50.0) |
| Female | 249 (69.9) | 261 (66.8) | 27 (73.0) | 8  (80.0) | 225 (71.9) | 304 (65.9) | 348 (68.1) | 185 (69.8) | 4  (50.0) |
| **Age** | 24.9±5.2 | 23.6±3.8 | 21.4±2.1 | 22.4±1.9 | 23.2±3.3 | 24.8±5.1 | 24.6±4.9 | 23.3±3.3 | 20.7±2.0 |
| **GPA** | 9.3±0.6 | 9.1±0.6 | 9.2±0.9 | 9.1±0.8 | 9.1±0.6 | 9.2±0.6 | 9.2±0.6 | 9.1±0.6 | 10.0±0.0 |
| **Study level** |  |  |  |  |  |  |  |  |  |
| I | 17  (4.8) | 62  (15.8) | 22  (59.5) | 4  (40.0) | 68  (21.7) | 29  (6.3) | 24  (4.7) | 71  (26.7) | 6  (75.0) |
| II-VI | 247  (69.6) | 277  (70.7) | 13  (35.1) | 6  (60.0) | 208 (66.2) | 323  (70.2) | 385 (75.5) | 150 (56.4) | 2  (25.0) |
| PhD | 91  (25.6) | 53  (13.5) | 2  (5.4) | 0  (0.0) | 38  (12.1) | 108  (23.5) | 101 (19.8) | 45  (16.9) | 0  (0.0) |
| **Scientific field of interest** | | | | | | | | | |
| Preventative/  Preclinical | 102 (44.3) | 95  (36.4) | 6  (54.5) | 1  (25.0) | 73  (36.5) | 129 (43.3) | 158 (43.6) | 45  (32.4) | 0  (0.0) |
| Clinical | 128 (55.7) | 166 (63.6) | 5  (45.5) | 3  (75.0) | 127 (63.5) | 169 (56.7) | 204 (56.4) | 94  (67.6) | 1 (100.0) |

*Favorable
