# Supplementary material for: Assessing attitudes toward research and plagiarism among medical students: a multi-site study
Source: Philos Ethics Humanit Med. 2024 Nov 15;19:11. doi: 10.1186/s13010-024-00161-z (PMC11566133; doi:10.1186/s13010-024-00161-z)
Supplement: Supplementary file 2 — Additional file 2. Participants’ responses to ATR questionnaire. [file 13010_2024_161_MOESM2_ESM.docx]

**Table** Participants’ responses to ATR questionnaire

| **Items, n (%)** | **1** | **2** | **3** | | **4** | | **5** | | **6** | **7** |
| --- | --- | --- | --- | --- | --- | --- | --- | --- | --- | --- |
| **Research usefulness** |  | | | | |  | | | | |
| Research is useful for my career | 9 (1.1) | 12 (1.5) | 23 (2.9) | | 70 (8.8) | | 62 (7.8) | | 224 (28.2) | 393 (49.6) |
| Research is connected to my field of study | 4 (0.5) | 13 (1.6) | 25 (3.2) | | 75 (9.5) | | 86 (10.8) | | 273 (34.4) | 317 (40.0) |
| Research should be indispensable in my professional training | 12 (1.5) | 27 (3.4) | 41 (5.2) | | 130 (16.4) | | 115 (14.5) | | 233 (29.4) | 235 (29.7) |
| Research should be taught to all students | 19 (2.4) | 23 (2.9) | 34 (4.3) | | 80 (10.1) | | 110 (13.9) | | 240 (31.4) | 278 (35.1) |
| Research is useful to every professional | 5 (0.6) | 10 (1.3) | 28 (3.5) | | 87 (11.0) | | 101 (12.7) | | 250 (31.5) | 312 (39.3) |
| Research is very valuable | 5 (0.6) | 10 (1.3) | 33 (4.2) | | 74 (9.3) | | 103 (13.0) | | 294 (37.1) | 274 (34.6) |
| I will employ research approaches in my profession | 9 (1.1) | 14 (1.8) | 24 (3.0) | | 99 (12.5) | | 117 (14.8) | | 282 (35.6) | 248 (31.3) |
| The skills I have acquired in research will be helpful to me in the future | 7 (0.9) | 12 (1.5) | 24 (3.0) | | 84 (10.6) | | 84 (10.6) | | 234 (29.5) | 348 (43.9) |
| Knowledge from research is as useful as writing | 5 (0.6) | 17 (2.1) | 30 (3.8) | | 82 (10.3) | | 102 (12.9) | | 245 (30.9) | 312 (39.3) |
| **Research anxiety** |  | | | | |  | | | | |
| Research makes me nervous* | 55 (6.9) | 115 (14.5) | 185 (23.3) | | 139 (17.5) | | 93 (11.7) | | 118 (14.9) | 88 (11.1) |
| Research is stressful* | 90 (11.3) | 195 (24.6) | 191 (24.1) | | 131 (16.5) | | 84 (10.6) | | 72 (9.1) | 30 (3.8) |
| Research makes me anxious* | 43 (5.4) | 23 (2.9) | 182 (23.0) | | 117 (14.8) | | 78 (9.8) | | 164 (20.7) | 121 (15.3) |
| Research scares me* | 32 (4.0) | 64 (8.1) | 143 (18.0) | | 127 (16.0) | | 91 (11.5) | | 195 (24.6) | 141 (17.8) |
| Research is a complex subject* | 115 (14.5) | 200 (25.2) | 177 (22.3) | | 172 (21.7) | | 69 (8.7) | | 48 (6.1) | 12 (1.5) |
| Research is complicated* | 66 (8.3) | 181 (22.8) | 243 (30.6) | | 153 (19.3) | | 82 (10.3) | | 53 (6.7) | 15 (1.9) |
| Research is difficult* | 75 (9.5) | 213 (26.9) | 206 (26.0) | | 142 (17.9) | | 76 (9.6) | | 61 (7.7) | 20 (2.5) |
| **Positive attitudes** |  | | |  | | | |  | | |
| I love research | 15 (1.9) | 13 (1.6) | 37 (4.7) | | 105 (13.2) | | 114 (14.4) | | 263 (33.2) | 246 (31.0) |
| I enjoy research | 13 (1.6) | 22 (2.8) | 38 (4.8) | | 106 (13.4) | | 148 (18.7) | | 278 (35.1) | 188 (23.7) |
| I like research | 11 (1.4) | 18 (2.3) | 22 (2.8) | | 78 (9.8) | | 90 (11.3) | | 297 (37.4) | 277 (34.9) |
| I am interested in research | 16 (2.0) | 21 (2.6) | 31 (3.9) | | 79 (10.0) | | 97 (12.2) | | 263 (33.2) | 286 (36.1) |
| Research is pleasant | 14 (1.8) | 25 (3.2) | 48 (6.1) | | 163 (20.6) | | 221 (27.9) | | 215 (27.1) | 107 (13.5) |
| Research is interesting | 12 (1.5) | 13 (1.6) | 24 (3.0) | | 69 (8.7) | | 88 (11.1) | | 315 (39.7) | 272 (34.3) |
| Most students benefit from research | 10 (1.3) | 24 (3.0) | 48 (6.1) | | 122 (15.4) | | 125 (15.8) | | 240 (30.2) | 224 (28.2) |
| I am inclined to study the details of research | 9 (1.1) | 21 (2.6) | 42 (5.3) | | 155 (19.5) | | 171 (21.6) | | 245 (30.9) | 150 (19.0) |
| **Relevance to life** |  | | | | |  | | | | |
| I use research in my daily life | 40 (5.0) | 100 (12.6) | 122 (15.4) | | 217 (27.4) | | 152 (19.2) | | 108 (13.6) | 54 (6.8) |
| Research-orientated thinking plays an important role in everyday life | 15 (1.9) | 34 (4.3) | 57 (7.2) | | 176 (22.2) | | 177 (22.3) | | 186 (23.4) | 148 (18.7) |
| Research thinking does not apply to my personal life* | 10 (1.3) | 50 (6.3) | 71 (9.0) | | 157 (19.8) | | 133 (16.8) | | 229 (28.9) | 143 (18.0) |
| Research is irrelevant to my life* | 13 (1.6) | 29 (3.6) | 28 (3.5) | | 88 (11.1) | | 96 (12.1) | | 272 (34.3) | 267 (33.7) |
| **Difficulty of research** | | | | | | | | | | |
| I find it difficult to understand the concepts of research* | 12 (1.5) | 23 (2.9) | 78 (9.8) | | 136 (17.2) | | 104 (13.1) | | 273 (34.4) | 167 (21.1) |
| I make many mistakes in research* | 19 (2.4) | 55 (6.8) | 135 (17.1) | | 205 (25.9) | | 130 (16.4) | | 175 (22.1) | 74 (9.3) |

*Recoded

1-Strongly disagree; 7-Strongly agree
