# Supplementary material for: Assessing attitudes toward research and plagiarism among medical students: a multi-site study
Source: Philos Ethics Humanit Med. 2024 Nov 15;19:11. doi: 10.1186/s13010-024-00161-z (PMC11566133; doi:10.1186/s13010-024-00161-z)
Supplement: Supplementary file 3 — Additional file 3. Participants’ responses to ATP questionnaire. [file 13010_2024_161_MOESM3_ESM.docx]

**Table** Participants’ responses to ATP questionnaire

| **Statement** | | **Strongly disagree**  **n (%)** | **Disagree**  **n (%)** | **Neither agree nor disagree**  **n (%)** | **Agree**  **n (%)** | | **Strongly agree**  **n (%)** |
| --- | --- | --- | --- | --- | --- | --- | --- |
| **Statements describing positive attitudes toward plagiarism** | |  |  |  |  | |  |
| 1. | Sometimes one cannot avoid using other people’s words without citing the source, because there are only so many ways to describe something. | 65 (8.2) | 122 (15.4) | 201 (25.3) | 302 (38.1) | | 103 (13.0) |
| 2. | It is justified to use previous descriptions of a method, because the method itself remains the same. | 84 (10.6) | 191 (24.1) | 248 (31.3) | 201 (25.3) | | 69 (8.7) |
| 3. | Self-plagiarism is not punishable because it is not harmful (one cannot steal from oneself). | 104 (13.1) | 151 (19.0) | 266 (33.5) | 152 (19.2) | | 120 (15.1) |
| 4. | Plagiarized parts of a paper may be ignored if the paper is of great scientific value. | 281 (35.4) | 258 (32.5) | 164 (20.7) | 71 (9.0) | | 19 (2.4) |
| 5. | Self-plagiarism should not be punishable in the same way as plagiarism is. | 74 (9.3) | 100 (12.6) | 241 (30.4) | 264 (33.3) | | 114 (14.4) |
| 6. | Young researchers who are just learning the ropes should receive milder punishment for plagiarism. | 190 (24.0) | 219 (27.6) | 210 (26.5) | 132 (16.6) | | 42 (5.3) |
| 7. | If one cannot write well in a foreign language (eg, English), it is justified to copy parts of a similar paper already published in that language. | 307 (38.7) | 276 (34.8) | 138 (17.4) | 46 (5.8) | | 26 (3.3) |
| 8. | I could not write a scientific paper without plagiarizing. | 387 (48.8) | 230 (29.0) | 120 (15.1) | 41 (5.2) | | 15 (1.9) |
| 9. | Short deadlines give me the right to plagiarize a bit. | 427 (53.8) | 206 (26.0) | 101 (12.7) | 36 (4.5) | | 23 (2.9) |
| 10. | When I do not know what to write, I translate a part of a paper from a foreign language. | 326 (41.1) | 222 (28.0) | 156 (19.7) | 67 (8.4) | | 22 (2.8) |
| 11. | It is justified to use one’s own previously published work without providing citation in order to complete the current work. | 248 (31.3) | 234 (29.5) | 204 (25.7) | 75 (9.5) | | 32 (4.0) |
| 12. | If a colleague of mine allows me to copy from her/his paper, I’m NOT doing anything bad, because I have his/her permission. | 297 (37.5) | 235 (29.6) | 157 (19.8) | 66 (8.3) | | 38 (4.8) |
| **Statements describing negative attitudes toward plagiarism** | |  |  |  |  | |  |
| 13. | Plagiarists do not belong in the scientific community. | 31 (3.9) | 57 (7.2) | 189 (23.8) | 296 (37.3) | | 220 (27.7) |
| 14. | The names of the authors who plagiarize should be disclosed to the scientific community. | 28 (3.5) | 93 (11.7) | 266 (33.5) | 248 (31.3) | | 158 (19.9) |
| 15. | In times of moral and ethical decline, it is important to discuss issues like plagiarism and self-plagiarism. | 15 (1.9) | 21 (2.6) | 104 (13.1) | 366 (42.4) | | 317 (40.0) |
| 16. | Plagiarizing is as bad as stealing an exam. | 34 (4.3) | 55 (6.9) | 171 (21.6) | 298 (37.6) | | 235 (29.6) |
| 17. | Plagiarism impoverishes the investigative spirit. | 21 (2.6) | 26 (3.3) | 125 (15.8) | 302 (38.1) | | 319 (40.2) |
| 18. | A plagiarized paper does no harm science. * | 37 (4.7) | 54 (6.8) | 115 (14.5) | 250 (31.5) | | 337 (42.5) |
| 19. | Since plagiarism is taking other people’s words rather than tangible assets; it should NOT be considered as a serious offense. * | 24 (3.0) | 44 (5.5) | 157 (19.8) | 278 (35.1) | | 290 (36.6) |
| **Statements describing subjective norms toward plagiarism** | |  |  |  | |  |  |
| 20. | Authors say they do NOT plagiarize, when in fact they do. | 42 (5.3) | 78 (9.8) | 445 (56.1) | 177 (22.3) | | 51 (6.4) |
| 21. | Those who say they have never plagiarized are lying. | 62 (7.8) | 146 (18.4) | 376 (47.4) | 141 (17.8) | | 68 (8.6) |
| 22. | Sometimes I’m tempted to plagiarize, because everyone else is doing it (students, researchers, physicians). | 199 (25.1) | 233 (29.4) | 229 (28.9) | 104 (13.1) | | 28 (3.5) |
| 23. | I keep plagiarizing because I haven’t been caught yet. | 466 (58.8) | 208 (26.2) | 96 (12.1) | 15 (1.9) | | 8 (1.0) |
| 24. | I work (study) in a plagiarism-free environment. | 27 (3.4) | 118 (14.9) | 357 (45.0) | 187 (23.6) | | 104 (13.1) |
| 25. | Plagiarism is not a big deal. | 293 (36.9) | 315 (39.7) | 149 (18.8) | 21 (2.6) | | 15 (1.9) |
| 26. | Sometimes I copy a sentence or two just to become inspired for further writing. | 208 (26.2) | 244 (30.8) | 211 (26.6) | 100 (12.6) | | 30 (3.8) |
| 27. | I don’t feel guilty for copying verbatim a sentence or two from my previous papers. | 234 (29.5) | 191 (24.1) | 230 (29.0) | 103 (13.0) | | 35 (4.4) |
| 28. | Plagiarism is justified if I currently have more important obligations or tasks to do. | 371 (46.8) | 256 (32.3) | 119 (15.0) | 34 (4.3) | | 13 (1.6) |
| 29. | Sometimes, it is necessary to plagiarize. | 266 (33.5) | 236 (29.8) | 201(25.3) | 66 (8.3) | | 24 (3.0) |

*Recoded
