# Supplementary material for: Assessing attitudes toward research and plagiarism among medical students: a multi-site study
Source: Philos Ethics Humanit Med. 2024 Nov 15;19:11. doi: 10.1186/s13010-024-00161-z (PMC11566133; doi:10.1186/s13010-024-00161-z)
Supplement: Supplementary file 4 — Additional file 4. Attitudes toward research and plagiarism among first year students. [file 13010_2024_161_MOESM4_ESM.docx]

**Table** Attitudes toward research and plagiarism among first year students

| **n=101** | **ATR** | | | | | | **ATP** | | |
| --- | --- | --- | --- | --- | --- | --- | --- | --- | --- |
|  | **Research usefulness** | **Research anxiety** | **Positive attitudes** | **Relevance to life** | **Difficulty of research** | **Total ATR** | **Positive attitudes** | **Negative attitudes** | **Subjective norms** |
| Gender |  |  |  |  |  |  |  |  |  |
| Male | 5.0±1.3 | 4.0±1.2 | 4.5±1.2 | 4.2±1.2 | 4.3±1.5 | 4.5±0.9 | 3.1±0.6 | 3.6±0.8 | 3.0±0.8 |
| Female | 4.7±1.5 | 4.1±1.3 | 4.0±1.6 | 4.2±1.0 | 4.2±1.6 | 4.3±0.9 | 3.2±0.8 | 3.3±0.8 | 3.0±0.8 |
| Age, r | 0.161 | -0.052 | 0.198* | 0.178 | 0.077 | 0.173 | 0.008 | 0.048 | -0.028 |

Data are presented as mean±sd;

r-Pearson correlation coefficient;

*p≤0.050
