# Supplementary material for: Assessing attitudes toward research and plagiarism among medical students: a multi-site study
Source: Philos Ethics Humanit Med. 2024 Nov 15;19:11. doi: 10.1186/s13010-024-00161-z (PMC11566133; doi:10.1186/s13010-024-00161-z)
Supplement: Supplementary file 5 — Additional file 5. Attitudes toward research and plagiarism among II-VI year students. [file 13010_2024_161_MOESM5_ESM.docx]

**Table** Attitudes toward research and plagiarism among II-VI year students

| **n=546** | **ATR** | | | | | | **ATP** | | |
| --- | --- | --- | --- | --- | --- | --- | --- | --- | --- |
|  | **Research usefulness** | **Research anxiety** | **Positive attitudes** | **Relevance to life** | **Difficulty of research** | **Total ATR** | **Positive attitudes** | **Negative attitudes** | **Subjective norms** |
| Gender |  |  |  |  |  |  |  |  |  |
| Male | 5.9±0.9 | 3.9±1.2 | 5.8±0.9 | 5.1±1.0 | 5.1±1.2 | 5.2±0.7 | 2.4±0.7 | 4.0±0.6 | 2.2±0.7 |
| Female | 5.9±1.0 | 3.6±1.2* | 5.7±1.0 | 5.0±0.9 | 5.0±1.2 | 5.1±0.7 | 2.4±0.7 | 3.9±0.7 | 2.2±0.7 |
| Age, r | -0.082 | 0.020 | -0.095* | -0.074 | -0.068 | -0.080 | -0.020 | -0.083 | 0.107* |
| GPA, r | 0.158** | 0.086* | 0.193** | 0.163** | 0.138* | 0.209** | -0.132* | 0.132* | -0.091* |
| Scientific field, (n=502) | | |  |  |  |  |  |  |  |
| Preventive/Preclinical | 6.0±1.0 | 3.7±1.2 | 5.8±1.0* | 5.1±0.9 | 5.2±1.1 | 5.2±0.7 | 2.3±0.7 | 4.0±0.7 | 2.1±0.6* |
| Clinical | 5.9±0.9 | 3.7±1.2 | 5.6±0.9 | 5.0±1.0 | 5.0±1.2 | 5.1±0.7 | 2.4±0.7 | 3.9±0.6 | 2.3±0.6 |

Data are presented as mean±sd;

r-Pearson correlation coefficient;

*p≤0.050; **p<0.001
