# Supplementary material for: Assessing attitudes toward research and plagiarism among medical students: a multi-site study
Source: Philos Ethics Humanit Med. 2024 Nov 15;19:11. doi: 10.1186/s13010-024-00161-z (PMC11566133; doi:10.1186/s13010-024-00161-z)
Supplement: Supplementary file 6 — Additional file 6. Attitudes toward research and plagiarism among PhD students. [file 13010_2024_161_MOESM6_ESM.docx]

**Table** Attitudes toward research and plagiarism among PhD students

| **n=146** | **ATR** | | | | | | **ATP** | | |
| --- | --- | --- | --- | --- | --- | --- | --- | --- | --- |
|  | **Research usefulness** | **Research anxiety** | **Positive attitudes** | **Relevance to life** | **Difficulty of research** | **Total ATR** | **Positive attitudes** | **Negative attitudes** | **Subjective norms** |
| Gender |  |  |  |  |  |  |  |  |  |
| Male | 5.8±1.0* | 3.6±1.1 | 5.6±1.0 | 5.1±1.1 | 4.8±1.4 | 5.1±0.7 | 2.4±0.6* | 4.1±0.5 | 2.4±0.7 |
| Female | 6.2±0.6 | 3.3±1.0 | 5.9±0.7 | 5.4±1.0 | 4.6±1.3 | 5.2±0.6 | 2.1±0.6 | 4.2±0.5 | 2.2±0.6 |
| Age, r | -0.111 | 0.043 | -0.088 | 0.080 | -0.022 | -0.040 | -0.172* | 0.146 | -0.135 |

Data are presented as mean±sd;

r-Pearson correlation coefficient;

*p≤0.050
