# Supplementary material for: Assessing attitudes toward research and plagiarism among medical students: a multi-site study
Source: Philos Ethics Humanit Med. 2024 Nov 15;19:11. doi: 10.1186/s13010-024-00161-z (PMC11566133; doi:10.1186/s13010-024-00161-z)
Supplement: Supplementary file 7 — Additional file 7. Univariate and multivariate linear regression analysis of undergraduate students who participated at students’ conference with ATR subscales as dependent variables. [file 13010_2024_161_MOESM7_ESM.docx]

**Table** Univariate and multivariate linear regression analysis of undergraduate students who participated at students’ conference with ATR subscales as dependent variables

| **ATR subscales**  n=502 | **Univariate** | | | **Multivariate** | | |
| --- | --- | --- | --- | --- | --- | --- |
|  | **β** | **t** | **p** | **β** | **t** | **p** |
| **Research usefulness** | |  |  |  |  |  |
| Gender, male | 0.029 | 0.649 | 0.517 |  |  |  |
| Age | -0.103 | 2.307 | **0.021** |  |  |  |
| GPA | 0.185 | 1.175 | **<0.001** | 0.181 | 4.083 | **<0.001** |
| Preclinical field | 0.033 | 0.733 | 0.464 |  |  |  |
| **Research anxiety** | |  |  |  |  |  |
| Gender, male | 0.076 | 1.705 | 0.089 |  |  |  |
| Age | 0.017 | 0.369 | 0.712 |  |  |  |
| GPA | 0.097 | 2.167 | **0.031** | 0.097 | 2.167 | **0.031** |
| Preclinical field | 0.011 | -0.242 | 0.809 |  |  |  |
| **Positive attitudes** | |  |  |  |  |  |
| Gender, male | 0.065 | 1.445 | 0.149 |  |  |  |
| Age | -0.111 | 2.487 | **0.013** |  |  |  |
| GPA | 0.209 | 4.761 | **<0.001** | 0.205 | 4.664 | **<0.001** |
| Preclinical field | 0.096 | 2.157 | **0.031** | 0.107 | 2.442 | **0.015** |
| **Relevance to life** | |  |  |  |  |  |
| Gender, male | 0.052 | 1.171 | 0.242 |  |  |  |
| Age | -0.082 | 1.843 | 0.066 |  |  |  |
| GPA | 0.169 | 3.813 | **<0.001** | 0.167 | 3.759 | **<0.001** |
| Preclinical field | 0.057 | 1.284 | 0.200 |  |  |  |
| **Difficulty of research** | | |  |  |  |  |
| Gender, male | 0.056 | 1.245 | 0.214 |  |  |  |
| Age | -0.075 | 1.682 | 0.093 |  |  |  |
| GPA | 0.147 | 3.303 | **0.001** | 0.145 | 3.257 | **0.001** |
| Preclinical field | 0.073 | 1.647 | 0.100 |  |  |  |
| **Total ATR** | |  |  |  |  |  |
| Gender, male | 0.080 | 1.787 | 0.075 |  |  |  |
| Age | -0.098 | 2.190 | **0.029** |  |  |  |
| GPA | 0.232 | 5.308 | **<0.001** | 0.229 | 5.232 | **<0.001** |
| Preclinical field | 0.070 | 1.575 | 0.116 |  |  |  |
