# Supplementary material for: Assessing attitudes toward research and plagiarism among medical students: a multi-site study
Source: Philos Ethics Humanit Med. 2024 Nov 15;19:11. doi: 10.1186/s13010-024-00161-z (PMC11566133; doi:10.1186/s13010-024-00161-z)
Supplement: Supplementary file 8 — Additional file 8. Univariate and multivariate logistic regression analysis of undergraduate students who participated at students’ conference with ATP domains as dependent variable. [file 13010_2024_161_MOESM8_ESM.docx]

**Table** Univariate and multivariate logistic regression analysis of undergraduate students who participated at students’ conference with ATP domains as dependent variable

| **Variable** | **Univariate** | | | **Multivariate** | | |
| --- | --- | --- | --- | --- | --- | --- |
|  | **p** | **OR** | **95%CI** | **p** | **OR** | **95%CI** |
| **Positive attitudes** |  |  |  |  |  |  |
| Gender | 0.761 | 1.058 | 0.735-1.523 |  |  |  |
| Age | 0.381 | 0.942 | 0.823-1.078 |  |  |  |
| GPA | **<0.001** | 1.762 | 1.305-2.379 |  |  |  |
| Scientific field | 0.101 | 1.349 | 0.943-1.930 |  |  |  |
| **Negative attitudes** |  |  |  |  |  |  |
| Gender | 0.103 | 1.369 | 0.939-1.997 |  |  |  |
| Age | 0.298 | 0.929 | 0.810-1.067 |  |  |  |
| GPA | **0.017** | 1.425 | 1.065-1.906 |  |  |  |
| Scientific field | 0.116 | 1.341 | 0.930-1.934 |  |  |  |
| **Subjective norms** |  |  |  |  |  |  |
| Gender | 0.301 | 1.243 | 0.823-1.876 |  |  |  |
| Age | 0.082 | 0.874 | 0.751-1.017 |  |  |  |
| GPA | **0.035** | 1.396 | 1.024-1.903 | **0.048** | 1.394 | 1.003-1.936 |
| Scientific field | **0.019** | 1.635 | 1.084-2.466 | **0.018** | 1.650 | 1.088-2.502 |
